# Quantification of the Underlying Mechanisms and Relationships Among Cancer, Metastasis, and Differentiation and Development

**DOI:** 10.3389/fgene.2019.01388

**Published:** 2020-03-02

**Authors:** Chong Yu, Qiong Liu, Cong Chen, Jin Wang

**Affiliations:** ^1^ State Key Laboratory of Electroanalytical Chemistry, Changchun Institute of Applied Chemistry, Chinese Academy of Sciences, Changchun, China; ^2^ University of Science and Technology of China, Hefei, China; ^3^ Department of Chemistry and of Physics and Astronomy, State University of New York at Stony Brook, Stony Brook, NY, United States

**Keywords:** landscape, kinetic path, cancer stem cell, epithelial mesenchymal transition, differentiation, metastasis

## Abstract

Recurrence and metastasis have been regarded as two of the greatest obstacles to cancer therapy. Cancer stem cells (CSCs) contribute to cancer development, with the distinctive features of recurrence and resistance to popular treatments such as drugs and chemotherapy. In addition, recent discoveries suggest that the epithelial mesenchymal transition (EMT) is an essential process in normal embryogenesis and tissue repair, as well as being a required step in cancer metastasis. Although there are many indications of the connections between metastasis and stem cells, these have often been studied separately or at most bi-laterally, not in an integrated way. In this study, we aimed to explore the global mechanisms and interrelationships among cancer, development, and metastasis, which are currently poorly understood. First, we constructed a core gene regulatory network containing specific genes and microRNAs of CSCs, EMT, and cancer. We uncovered seven distinct states emerging from the underlying landscape, denoted normal, premalignant, cancer, stem cell, CSC, lesion, and hyperplasia. Given the biological definition of each state, we also discuss the metastasis ability of each state. We show how and which types of cells can be transformed to a cancer state, and the connections among cancer, CSCs, and EMT. The barrier height and flux of the kinetic paths are explored to quantify how and which cells switch stochastically between the states. Our landscape model provides a quantitative approach to reveal the global mechanisms of cancer, development, and metastasis.

## Introduction

Cell phenotypes change during the development of cellular differentiation (Wang et al., 2011; Xu et al., 2014). Differentiation starts from an oosperm, which develops into a complex biont system and continues into adulthood as stem cells (SCs) divide and generate differentiated daughter cells during tissue repair and cell regeneration (Sell, 2004). Induced pluripotent SCs (iPS) provide an opportunity for therapeutic use (Takahashi et al., 2007).

Adult cells were reprogrammed into pluripotent SCs in 2006 (Takahashi and Yamanaka, 2006). This was a significant step in SC and regenerative biology, as the cell type switching could skip many intermediate steps. This lineage reprogramming technology may also have profound implications for cancer biology.

Cancer is one of the most deadly diseases in humans. Studies show that there are multiple factors associated with recurrence and metastasis. Moreover, cancer is fatal mainly owing to metastasis (Cowin et al., 2005). Many studies have focused on the genetic origins of cancer (Muller and Vousden, 2013; Martincorena and Campbell, 2015). The accumulation of mutations leads to malignant transformation, which has been described as a disease of clonal evolution. Through such mutation and selection, cells acquire the hallmarks of cancer (Lynch et al., 1998; Hanahan and Weinberg, 2000). Some cells may acquire hypoxic and fast-growing characteristics, or may develop new blood vessels and so on. This is a widely accepted aspect of the generation of cancer. On the other hand, many observations have demonstrated that cancer could be thought of as an intrinsic state which emerges from underlying gene regulation networks (Kauffman, 1971; Spano et al., 2012), which control a series of cellular activities and biological processes. The network can provide regulatory instructions which may affect early events of cancer (Blancafort et al., 2013). These network environmental and epigenetic effects can result in not only silencing of tumor suppressors but also reactivation of the silenced regions, which could prime subsequent events in the development of cancer (Liu et al., 2008; Rodrguez-Paredes and Esteller, 2011).

Cancer SCs (CSCs) can be defined as cells with the characteristics of cancerousness and stemness (Tang, 2012). Although cancer cells might be killed during chemotherapy or immune surveillance, CSCs can survive as “seeds” of the cancer (Hanahan and Coussens, 2012; Kreso et al., 2013), explaining the recurrence of cancer after treatment. Although the CSC theory was reported as early as 1952 (Hewitt, 1952), its importance has only recently been understood. CSCs have been shown to serve as the basis of cancer development, maintenance, metastasis, and recurrence (Dragu et al., 2015). In general, the differentiation and development process is due to primary SCs, and reprogramming is vice versa; this is important in tissue reengineering. Cellular reprogramming involves iPS, indicating the possibility of cell fate switching and transformation (Kondo and Raff., 2000). However, reprogramming often results in a cancer state, resulting in the transformed progenitors acquiring self-renewal and cancerous characteristics (Pavlova and Thompson, 2016). Furthermore, CSCs facilitate the primary tumor cells to migrate from one location to another, which is a key step in the metastatic cascade.

Epithelial mesenchymal transition (EMT) is an essential process through which most adult tissues maintain their migratory capacity in normal embryogenesis, wound healing, and tissue repair (Morel et al., 2012). CSCs can also implant into another organ through the EMT process (Wang et al., 2015). In EMT, a set of transcription factors (TFs) induce the early steps of metastasis (Scheel and Weinberg, 2012). Through EMT-TFs, differentiated epithelial cells can obtain mesenchymal traits to colonize foreign tissues and create new tumor sites in distant organs. Moreover, the EMT process is also the means by which non-SCs are transformed into SC states. Experiments have shown that inducing an EMT process during normal mammary epithelial cell differentiation can cause generation of mammary epithelial stem-like cells (Mani et al., 2008). This kind of experimental phenomena can be observed in both normal and cancerous tissues (Morel et al., 2008). Thereby, EMT is an important process which not only contributes to creating metastatic CSCs but also has a close relationship with CSCs (Chaffer et al., 2011).

Despite many results indicating the connections between metastasis and CSCs, or cancer and differentiation and development (Li and Wang, 2015), cancer, metastasis, and differentiation and development are rarely studied in an integrated way. Here, we aim to explore the connections among cancer, differentiation and development, and metastasis in a systematic and quantitative way. We start by constructing a core gene regulation network. In order to characterize the key points of the dynamic process, some specific genes and microRNAs of cancer, CSC, and EMT are included. In this work, we quantify the underlying landscape of cancer, metastasis, and differentiation and development. Furthermore, we include regulatory binding and unbinding information to make the model more precise. Seven states emerge from the landscape, which are quantified by the basins of attractions representing the normal, premalignant, cancer, SC, CSC, lesion, and hyperplasia states. In certain previous studies (Yu and Wang, 2016), normal, premalignant and cancer states were explored. In another model (Li and Wang, 2015), normal, cancer, CSC, and stem cell states were found. The lesion and hyperplasia states were not found in the previous theoretical studies but were observed in the experiments. They are predicted in our studies. We define these states by gene expression levels and biological significance. We also discuss the metastatic ability of these states. There are three pathways from the normal to the cancer state. Two kinetic paths which connect CSC state show the formation of cancer SCs from two sources. The optimal paths and barrier heights between the states illustrate how and which cells will be able to transform into the cancer state, and why cancer is so difficult to cure. This leads to a quantitative understanding of the degree of difficulty in curing the cancer. Moreover, the quantified landscape provides a portrait of the dynamic interrelationships among the biological processes of CSCs, EMT, and cancer. Finally, we use global sensitivity analysis to explore which regulatory process is more relevant to cancer therapy, which may provide guidance for future clinical experiments. This work helps to elucidate the origins of cancer, as well as the processes of differentiation and development in cancer and metastasis. This has clear clinical significance in understanding the role of CSCs in treatment response, therapeutic resistance, and cancer relapse.

## Results and Discussion

### Model Construction

**Figure 1:**
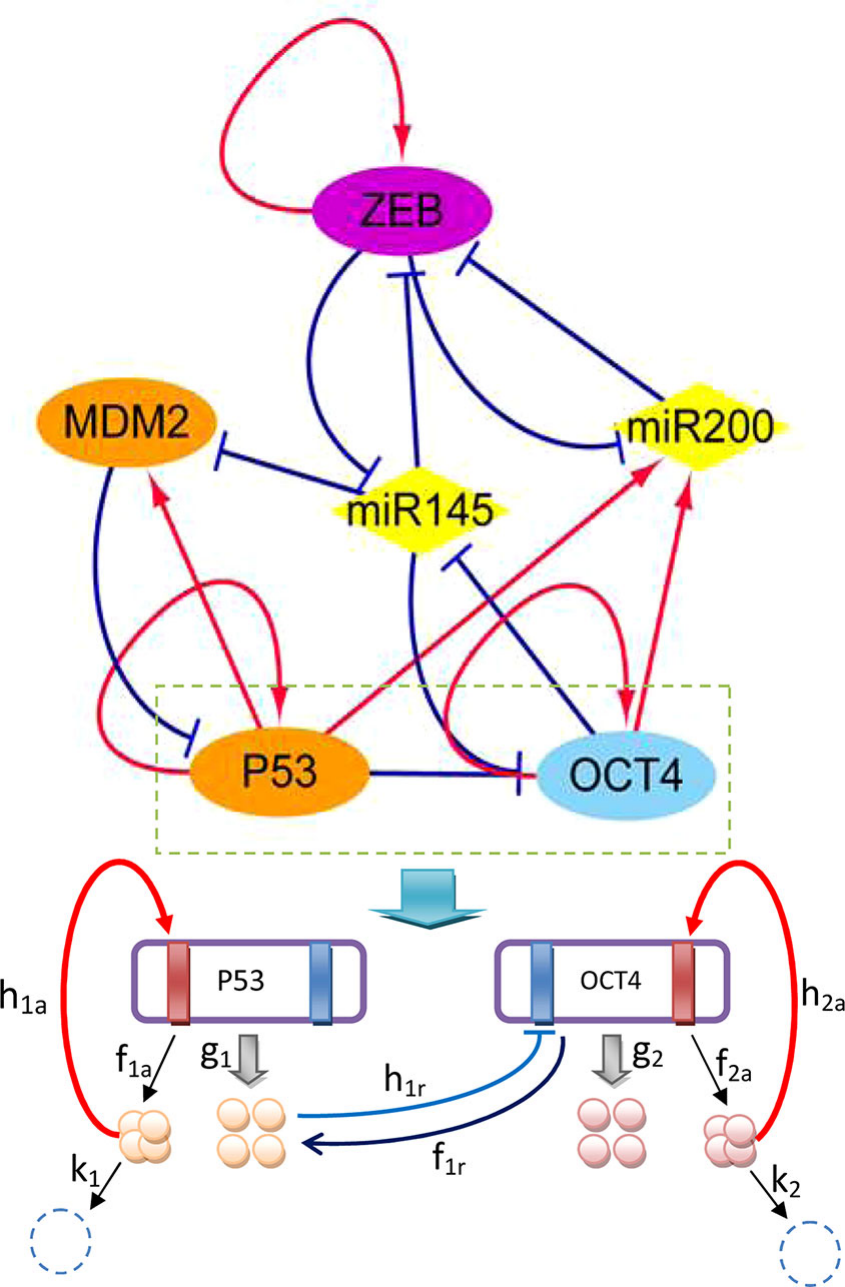
Diagram of the core gene regulatory network containing six nodes and 16 regulations (seven activations and nine repressions; arrows represent activating regulations, short bars represent repressing regulations). Diamond-shaped nodes represent microRNAs. Round orange nodes represent specific cancer genes, violet node represents the specific EMT gene, blue represents the specific CSC gene. Parameters: *k*_1_ = *k*_2_ = 1, λ*_a_* = 8, λ*_r_* = 0.5, *h_a_* = 2, *h_r_* = 1.875, *g*_0_ = 50, *f = k* ∗ *ω*.

To emphasize the characteristics of CSCs, EMT, and cancer, a core gene regulatory network was constructed to cover specific genes and microRNAs of the three aspects, as shown in **Figure 1**. MDM2 is an oncogene of cancer, P53 is a well-known tumor suppressor gene (Yu and Wang, 2016), ZEB is an EMT activator gene which suppresses the stemness-inhibition of a microRNA (mir-200) (Wellner et al., 2009), OCT4 is an essential gene which mediates phenotype self-renewal and stemness (Kumar et al., 2012), and mir-145 and mir-200 are two important microRNAs with vital roles in both CSC and EMT regulation (Liu et al., 2015). The arrows represent activation and the short bars represent repression. The details of the regulatory network and gene function can be seen in **Tables S1** and **S2** in the **Supporting Information**.

The network includes ZEB−|mir-145−|OCT4 and OCT4−|mir-145−|ZEB regulation, indicating that ZEB effectively activates (→) OCT4, while OCT4 effectively activates (→) ZEB. Therefore, stemness and metastasis promote each other: stemness can induce metastasis, and metastasis can also induce stemness. The regulation of OCT4−|mir-145−|MDM2 and MDM2−|P53−|OCT4 and ZEB−|mir-145−|MDM2 indicates that OCT4 effectively activates (→) MDM2, while MDM2 effectively activates (MDM2→) OCT4, and ZEB effectively activates (ZEB→) MDM2. MDM2 is known to be an oncogene. Therefore, stemness and metastasis can induce cancer, and cancer can also induce stemness. The regulation of ZEB−|mir-145−|MDM2 and MDM2−|P53→mir145−|ZEB also shows that ZEB effectively activates (→) MDM2, and MDM2 effectively activates (→) ZEB. Thus, metastasis and cancer can promote each other. From the network wiring, we can gain certain information about the reinforcing relationships among stemness, cancer, and metastasis.

### Methods

In our previous study (Yu and Wang, 2016), as the strong interaction, proteins and genes are treated as the same identity. We used differential equations to describe the gene regulatory network. The parameters are activation, repression and degradation items which describe the activation regulation rate, repression regulation rate and self-degradation rate, respectively. In this study, we use the chemical reactions to describe not only the protein concentration dynamics but also explicitly the underlying gene regulations (protein binding to the genes) and defined a set of rate parameters for each reaction to describe the gene regulatory process, which is stochastic. The underlying chemical reactions of gene regulation can be described as follows:

(1) $$G_{1}^{0\alpha \beta }+(n+1)P_{1}\overset{h_{1}}{\underset{f_{1}}{⇌}}G_{1}^{1\alpha \beta }+(n)P_{1}$$

(2) $$G_{1}^{\alpha 0\beta }+(n+2)P_{2}\overset{h_{2}}{\underset{f_{2}}{⇌}}G_{1}^{\alpha 1\beta }+(n)P_{2}$$

(3) $$G_{1}^{\alpha \beta 0}+(n+4)P_{3}\overset{h_{3}}{\underset{f_{3}}{⇌}}G_{1}^{\alpha \beta 1}+(n)P_{3}$$

(4) $$(n)G_{1}\overset{g}{\underset{k}{⇌}}(n+1)G_{1},$$

where *𝒢*_1_ represents a gene with three binding sites, 0 indicates the binding site which is unoccupied, and 1 indicates the binding site which is occupied. In the chemical reactions, the first binding site of *𝒢*_1_ can be occupied by a monomer, the second binding site of *𝒢*_1_ can be occupied by a dimer, and the third binding site can be occupied by a tetramer. *P_i_* (*i* = 1,2,3) represents the type of the protein regulator. The parameter in front of the protein, *P_i_*, represents its molecular number. The parameter *g* represents the protein synthesis rate and *k* represents the protein degradation rate; *h* represents the binding rate and *f* is the unbinding rate of regulatory proteins to the target genes.

In **Figure 1**, we take gene regulation of P53 and OCT4 as an example to illustrate the regulatory process. The red rectangles indicate activated binding sites for the genes, while the blue rectangles are repressed binding sites. P53 and OCT4 have protein synthesis rates of *g*_1_ and *g*_2_, and protein degradation rates of *k*_1_ and *k*_2_, respectively. The P53 and OCT4 proteins have binding rates of *h*_1_ *_a_* (*a* represents activation) and *h*_2_ *_a_* to their own activated binding sites, and unbinding rates of *f*_1_ *_a_* and *f*_2_ *_a_* from the binding sites. The P53 protein has a rate of *h*_1_ *_r_* (*r* represents repression) for binding to the repressing binding site of gene OCT4, and a rate of *f*_1_ *_r _* for unbinding from the binding site of OCT4.

For the first reaction (monomer binding site), the binding rate is given as *h*_1_ = *h*_1_ *n*_1_. For the second reaction (dimer binding site), the binding rate is given as *h*_1_ = *h*_1_ *n*_2_ (*n*_2_ −1)*/*2. For the third reaction (tetramer binding site), the binding rate is given as *h*_1_ = *h*_1_ *n*_3_(*n*_3_−1)(*n*_3_−2)(*n*_3_−3)*/*6. The protein synthesis rate is influenced by the regulated molecular number and regulated type. There are two regulated types: binding state and unbinding state. If the gene has *n* binding sites, it can give rise to 2*^n^* synthesis rates. The synthesis rate can be increased by a factor of λ*_a_* (*a* represents activation) or decreased by a factor of λ*_r_* (*r* represents repression). If there are two binding sites, one for activation and the other for repression, the four synthesis rates are set as: *g*_00_, *g*_01_ = *g*_00_λ*_a_*, *g*_10_ = *g*_00_λ*_r_*, and *g*_11_ = *g*_00_λ*_a_*λ*_r_*. We define the equilibrium constant *X_eq_ = f/h* and the adiabatic parameter *ω* = *f/k*. The latter is used to quantify the ratio of the unbinding rate of a protein to the gene and its degradation rate. If the value of *ω* is large, the regulatory processes are relatively fast compared with synthesis and degradation; this is sometimes termed adiabatic. If the value of *ω* is small, it means the regulatory processes are relatively slow. In this model, we set the parameters *k* = 1, *g*_0_ = 50, λ*_a_* = 8, λ*_r_* = 0.5, *h_a_* = 2, and *h_r_* = 1.875. If the protein switches on and off to the target gene relatively slowly, then the regulation process is non-adiabatic. In this work, we mainly focus on fast binding and unbinding, that is, the adiabatic case (*ω* = 1000).

In the adiabatic case, the stochastic reactions can be described by a master equation (Gillespie, 2000), with a probability *P*(*x,t*) of reaching the state *x* of the system at time *t*. The transition rates *M*(*x*|*x*′) are given as a matrix, for a system changing from state *x* to state *x*′, where

$$M(x|x^{\prime })=\{\begin{array}{ll} \geq 0, & if\;x\neq x^{\prime }\\ -\Sigma_{x\neq x^{\prime }}M(x|x^{\prime }), & if\;x=x^{\prime } \end{array}$$

The master equation can be expressed as the rate of change of *P*(*x,t*) for the combinations of possible transitions of *x*:

(5) $$\partial P/\partial t=\sum_{x\in X}M(x|x^{\prime })P(x,t)$$

The master equation can be further written in a more explicit form as:

(6) $$\partial P/\partial t=(M_{0}+M_{b})P,$$

where the probability *P* is a state vector. Each component of *P* represents the probability of the system with a protein number at a gene state. *M*_0_ is the diagonal part of the matrix *M* that represents the protein synthesis and degradation processes. *M_b_* is the non-diagonal part of the matrix *M* which represents the binding and unbinding regulation reactions. The binding and unbinding parts represent the reactions between gene states. The potential landscape of the gene regulation system can be defined as *U* = −ln*P* (Wang et al., 2008) . In practice, we use Gillespie algorithm (Gillespie et al., 1977) to simulate the gene regulatory network and effectively solve the master equation (see details in the **Supporting Information**).

### Definition and Metastatic Ability of Each Steady State and the Kinetic Paths of the Landscape

There are six nodes in our network. As it is difficult to visualize a six-dimensional space, we chose to discuss three specific genes, P53, ZEB, and OCT4, reflecting the cancer, EMT, and differentiation and development (with CSCs) aspects. P53 is a tumor suppressor gene. Normally functioning cells often have high gene expression levels of P53. Low gene expression of P53 is a general characteristic of cancer (Yang et al., 2013; Yu and Wang, 2016). OCT4 is a signature gene of SCs. Many studies have shown that OCT4 is critically involved in self-renewal and is a critical gene for cell differentiation and reprogramming (Lin et al., 2012). High gene expression of OCT4 indicates that cells have self-renewing ability, multi-differentiating potential, and strong proliferative ability. ZEB is a critical gene of the EMT process. The expression of ZEB can activate EMT, which is a required step in metastasis (Lamouille et al., 2014). The gene expression level of ZEB is a metastatic signature.

**Figure 2:**
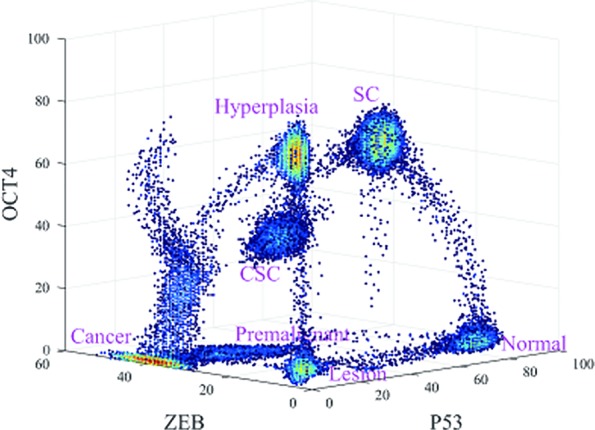
Three-dimensional landscape showing the normal, premalignant, cancer, SC, Cancer stem cell (CSC), lesion, and hyperplasia states, and optimal paths among these states.

As shown in **Figure 2**, seven states emerge, which are denoted normal, premalignant, cancer, SC, CSC, lesion, and hyperplasia. In the normal state, the gene expression level of P53 is high, and those of OCT4 and ZEB are low. Thus, if cells remain in the normal state, they maintain normal function and do not have the characteristics of SCs such as self-renewal or reprogramming, nor do they have metastasis ability. This is consistent with the regulation in the network, in which OCT4 and ZEB effectively activate each other and both repress P53 (ZEB−|mir-145−|OCT4, OCT4−|mir-145−|ZEB; this is because OCT4 and ZEB activate each other. ZEB−|mir-145−|MDM2−|P53 can be seen as ZEB−|P53, and OCT4−|mir-145−|MDM2−|P53 can be seen as OCT4−|P53). Therefore, OCT4 and ZEB can both have low expression. In this case, there is no further repression of P53, resulting in high P53 expression. Overall, the gene expression levels indicate that the cells in the normal state are in a healthy condition without metastasis or self-renewal capability.

The lesion state involves low expression levels of P53, OCT4, and ZEB. Low gene expression levels of OCT4 and ZEB indicate that the cells do not have the characteristics of SCs or metastatic ability. Low gene expression levels of P53 indicate that the cells do not have normal function, which may be caused by inflammation, pH, hypoxia, and so on (Jeremy et al., 2003). This is again consistent with the gene regulatory wiring. Mutual effective activation can give rise to a low expression state for both OCT4 and ZEB, as discussed earlier. However, MDM2 represses P53 and P53 activates MDM2. It is possible for MDM2 expression to be high while P53 expression is low, or for MDM2 expression to be low while P53 expression is high. The former corresponds to the lesion state, while the latter corresponds to a normal cell state. In this case, the gene expression levels indicate that the cells are not in a healthy condition but do not have metastatic or self-renewal capability.

In the hyperplasia state, compared with the lesion state, the expression level of OCT4 is high and the expression levels of P53 and ZEB are low. A high gene expression level of OCT4 implies that the cells have the characteristics of SCs, such as self-renewal or reprogramming. The hyperplasia state can be seen as the accumulation of cell damage, while the tissues which are inflamed start the self-repair process which helps to produce new cells to replace the pathological cells. In this process, OCT4 is also a significant player in self-repair and DNA replication (Rizzino, 2013). Low gene expression levels of ZEB indicate that the metastasis is not significant. A low expression level of P53 indicates that the cells are still in an abnormal condition. This is consistent with the gene regulatory wiring. High OCT4 expression can repress P53 expression, maintaining the low expression of P53. OCT4 can also repress ZEB by another route to keep ZEB expression levels low (OCT4→mir200−|ZEB, the same as the SC state). OCT4 can self-activate to keep its expression high. The gene expression levels indicate that the cells in the hyperplasia state are not in a healthy condition and have strong self-renewal but not metastatic capability. We considered the hyperplasia state to be a tumor state without metastasis. Cells in the lesion and hyperplasia states both have a degree of damage, as the gene expression levels of P53 are low. In general, they can be reversed to a normal state by the self-healing system, as the expression level of ZEB is low, indicating that metastasis has not yet started. Tumors exist mainly in the hyperplasia state according to our definition, in which they have certain hallmarks of cancer such as overgrowth in some organs. However, tumors are not fatal until they are metastatic. When the tumor is already in a metastatic condition, it is considered to be cancer.

In the cancer state, the gene expression level of ZEB is high, and the gene expression levels of OCT4 and P53 are low. For cells in the cancer state, the tumor suppressor gene P53 shows low expression levels, but the metastatic ability is obvious (high gene expression level of ZEB). Moreover, cancer cells in the terminally differentiated stage have lost the ability to proliferate or to alter their destiny; their stemness ability is relatively low as well. Thus, the gene expression level of OCT4 is low. That is consistent with the gene regulatory wiring. High ZEB expression will repress P53 so as to keep P53 at a low expression level. OCT4 expression may be low owing to self-degradation, despite effective activation by ZEB. The low expression levels of P53 and OCT4 mean they cannot effectively repress ZEB by another regulation route, so ZEB expression remains at a high level. The gene expression levels indicate that the cells in the cancer state have very significant cancerous characteristics and metastatic capability. Thus, the cancer state represents a tumor with metastasis.

The premalignant state is a transition state between the normal and cancer states. In the premalignant state, the expression level of P53 decreases and that of ZEB increases when the cells transform from the normal to the cancer state, that is, the cancerization and metastasis become increasingly significant. The metastatic ability of the premalignant state is intermediate, bridging those of the normal state and the complete cancer/metastasis state. Moreover, the intermediate expression level of ZEB indicated that the EMT is also in an intermediate state, which known as the partial (hybrid) epithelial/mesenchymal (E/M) state (Kumar Jolly, 2015; Pastushenko et al., 2018). The partial EMT can be considered as primary bad actors of metastases. This is consistent with the gene regulatory wiring. Relatively higher ZEB expression will repress P53 so as to keep P53 expression at a relatively low level. OCT4 expression may be low owing to self-degradation despite effective activation by ZEB. The relatively lower expression of P53 and low expression of OCT4 mean that they cannot effectively repress ZEB by another regulatory route, so ZEB expression remains at a relatively high level. The gene expression levels indicate that cells in the premalignant state have certain cancerous characteristics, partial EMT phenotype and metastatic capability. Therefore, we considered the premalignant state to represent tumors with a certain level of metastasis.

In the SC state, the gene expression levels of P53 and OCT4 are high, and that of ZEB is low. Cells in this state have stemness activity, so the expression levels of OCT4 are high. The expression level of P53 is also high and that of ZEB is low, indicating that the cells are functioning normally and their metastatic ability is not active. This is consistent with the regulatory wiring. When both OCT4 and ZEB have high expression as a result of their effective mutual activation, their repression leads to low P53 expression. When the expression of ZEB is low, its repression of P53 is weak, leading to high expression of P53, which represses OCT4. However, OCT4 can sustain its high level of expression through self-activation. OCT4 is involved in an alternative route (OCT4→mir200−|ZEB); the protein concentration determines which path is dominant. If the concentration of mir-200 is dominant, the route is repression. This route can repress ZEB and keep its expression levels low. The gene expression levels indicate that the cells in the SC state are in a healthy condition with strong self-renewal capability, but without the metastatic capability.

In the CSC state, the expression levels of P53, OCT4, and ZEB are all intermediate. These cells are in a transition between the SC and the cancer state. CSCs show some characteristics of cancerization and self-renewal (stemness), as their gene expression level of P53 is lower and that of OCT4 is higher than in the normal state. Moreover, the elevated gene expression of ZEB indicates that the cells have a certain metastatic ability and an intermediate EMT phenotype, in between those of the SC and cancer state. Many studies suggest that partial EMT associates with Stemness. Cells in partial EMT state are most likely to gain stemness (Strauss et al., 2011; Kumar Jolly et al., 2014; Grosse-Wilde et al., 2015).This is consistent with the regulatory wiring. When both OCT4 and ZEB have high expression levels as a result of their effective mutual activation, their repression leads to low P53 expression. The gene expression levels indicate that cells in the CSC state have certain cancerous characteristics, self-renewal capability, a partial EMT phenotype and metastatic ability. Therefore, the CSC state represents a tumor with a certain degree of stemness and metastasis.

We also compared our landscape with the experimental data. To quantify the landscape from the experimental results, we project the data to 3 dimensions in expression levels of P53, ZEB, and OCT4. This projection for the landscape can be used to reflect the characteristic features of the cancer, EMT, and differentiation/development (with CSCs). The RNA-seq data can only reflect the gene expressions at the transcriptional level. The protein concentrations reflect the gene expressions at the post translational level. Due to the post transcriptional and post translational influences, the RNA-seq data may not be able to completely determine the activities of these genes.

**Figure 3:**
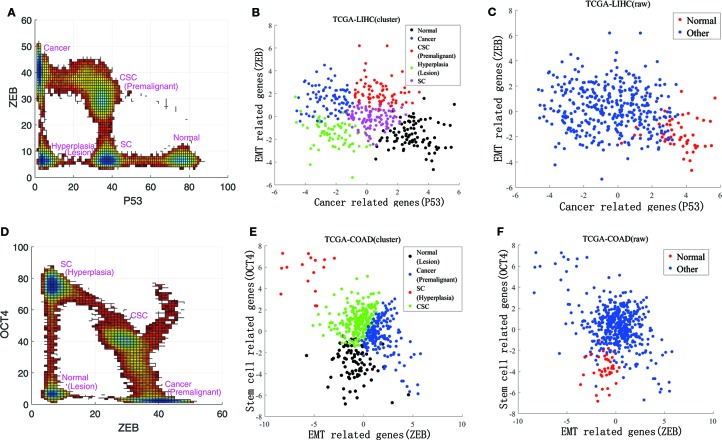
Comparisons of experimental data and the steady states of our landscape. **(A)** is our landscape projection to X and Y axes. **(B**, **C)** show the data clustering and raw data for LIHC. **(D)** is our landscape projection to Y and Z axes. **(E**, **F)** show the data clustering and raw data for COAD.

Instead of directly using the individual gene RNA-seq data, we consider some other genes which are regulated by or indirectly regulated by the individual gene (P53 representing the cancer group for example). The downstream genes transcription levels (18 of them related to P53 in this example) are determined by the upstream genes protein activities (post translation level). Therefore, these 18 genes are also cancer related genes and their genes transcriptional data can be used to reflect p53s post translation level gene expressions in some respect. This can lead to more complete information on P53 gene expressions at the post translation level beyond the transcription level which is crucial for describing the function. It serves as the rational for choosing more genes (total of 40) instead of individual genes (six genes) we focused on at the beginning of the analysis. We then analyzed these three groups of experimental data using principal component analysis. By selecting the first principal component for each group, respectively, the RNA-seq data could be reduced to three dimensions. In **Figures 3B, C, E**, and **F**, RNA-seq data are represented by their first principal component. **Figure 3A** shows our landscape projection to the X and Y axes. As the CSC state coincided with the premalignant state, and the hyperplasia state coincided with the lesion state, there were five steady states: normal, CSC (premalignant), cancer, hyperplasia (lesion), and SC. In **Figure 3B**, there are five clusters which correspond to the five states. **Figure 3D** shows our landscape projection to the Y and Z axes. As the normal state coincided with the lesion state, the hyperplasia state coincided with the SC state, and the cancer state was connected with the premalignant state, there were four steady states: normal (lesion), cancer (premalignant), CSC, and SC (hyperplasia). In **Figure 3E**, there are four clusters which correspond to the four states. **Figures 3C**, **F** show the raw data for LIHC and COAD, which were used to validate the clustering results in **Figures 3B**, **E**. The normal state positions of the cluster results coincide with those of the raw data.

From a landscape perspective, there were several major kinetic paths that could be quantitatively explored. When the expression level of ZEB increases, the paths from the SC to CSC and the CSC to cancer states become prominent. These two paths show that the formation of CSCs has two main sources. One route of CSC generation involves somatic SCs with self-renewal capabilities; these have the potential to divide into both SCs and specialized somatic cells, which are destined to stop proliferating or die (Lobo et al., 2007). If these SCs are out of control with respect to stopping division, but still keep their self-renewal and differentiation abilities, they become CSCs (Ponti et al., 2005; Ye and Weinberg, 2015). Another route for generating CSCs exists owing to a minor proportion of cancer cells with the capacity for self-renewal and differentiation in their progeny (Liu et al., 2015). Many experiments have demonstrated that terminally differentiated cancer cells can gain SC properties under specific epigenetic conditions (Tang, 2012). These SC-like cancer cells drive cell growth and metastasis, and are considered to be CSCs. Many reports have shown that cancer cells undergoing EMT can obtain SC-like characteristics (Mani et al., 2008), demonstrating the connection between EMT and CSC. These have been found in hematopoietic (Bonnet and Dick, 1997) and solid tumors such as brain (Singh et al., 2004) and breast cancers (Alhajj et al., 2003). These two paths driving CSC generation lead independently to the capacity for self-renewal, differentiation, and migration. The kinetic paths in the landscape view illustrate the dynamic transitions of SCs, CSCs, and cancer. Owing to these diversifications, CSCs present a major challenge with respect to drug resistance and cancer recurrence.

The landscape view also shows that there is more than one pathway from the normal to the cancer state. There are at least three major paths. The first is from the SC to CSC to cancer state. Stem cells can gain cancer characteristics and become CSCs. Recently, some studies tracing CD133+ cells have provided direct evidence that SCs are susceptible to cancerous transformation (Medema, 2013; Zhu et al., 2016). CSCs inherit many characteristics of SCs, including self-renewal and differentiation. Moreover, CSCs have cancerization characteristics such as uncontrollable growth and metastasis. CSCs can be asymmetrically divided into cancer cells and CSCs (Sell, 2004). Thus, CSCs can be seen as the seeds of cancer cells. This path involves both stemness through CSCs and the metastasis (or EMT) process (half-metastasis state for CSCs). When cells are in the SC state, the gene expression levels of P53 and OCT4 are high, but that of ZEB low. This indicates that the cells are in a healthy condition and have stemness but not the metastatic feature. When the cells transform to the CSC state, the gene expression levels of P53 and OCT4 both decrease, and that of ZEB increases. This indicates that the cells in the CSC state become cancerous and have a certain metastatic ability.

The second pathway is from the normal to premalignant to cancer state. This can be seen as a cancerous process. On this path, the gene expression level of P53 decreases and that of ZEB increases. This indicates that the cells not only show a trend of pathological changes but also have metastatic characteristics. This path involves metastasis or EMT, since the premalignant state is a half-cancer and half-metastasis. The cells in the normal state are in a healthy condition and do not have the metastatic or stemness features, as the gene expression level of P53 is relatively high but those of OCT4 and ZEB are low. When the cells transform to a premalignant state, the gene expression level of P53 decreases and that of ZEB increases. This indicates that the cells exhibit half-cancerous and half-metastatic features. When the cells are in the cancer state, the gene expression level of P53 is low and that of ZEB is high. OCT4 gene expression is also low. This indicates that the cells in the cancer state gain a strong metastatic ability and are differentiated without stemness.

The third pathway is from the normal to lesion to hyperplasia to cancer state. This can be seen as a process by which normal cells develop into the lesion state, gaining proliferation ability (hyperplasia), then turning malignant and eventually achieving the cancer state. Some experiments have shown that a lesion often occurred before the hyperproliferative changes (Jeremy et al., 2003). Hyperplasia is accumulated to a certain degree; cells possess metastatic ability and ultimately transform to the cancer state. This pathway involves stemness and the EMT process during its last stage. The cells in the lesion state are not in a healthy condition and do not have the stemness or metastasis features, as the gene expression level of P53, ZEB, and OCT4 are low. When the cells are in the hyperplasia state, the gene expression level of OCT4 becomes high, although the expression levels of the other two genes do not change significantly. This indicates that the cells in the hyperplasia state have significant stemness features, as cell damage induces their self-renewal ability to enable self-repair (Rizzino, 2013). However, the gene expression level of ZEB remains low. This indicates that cells in the hyperplasia state do not have the metastatic feature. When the cells reach the cancer state, the gene expression level of ZEB becomes very high and metastasis is obvious. These three paths address a central question in cancer biology: how and which cells can be transformed to cancer. These results also indicate that cancer is difficult to cure because the formation of these paths.

### Barrier Heights and Flux of Kinetic Paths

**Figure 4:**
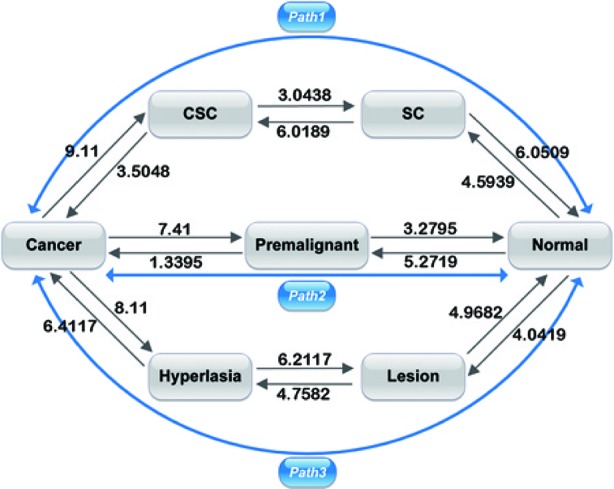
Barrier heights between normal, premalignant, cancer, SC, Cancer stem cell (CSC), lesion, and hyperplasia states, and optimal paths among these states. Black arrows represent the barrier from one state to another. The data marked represent the barrier height to overcome. Blue arrows represent the kinetic paths from normal to cancer state and the reverse.

**Figure 4** shows the barrier heights between the normal, premalignant, cancer, SC, CSC, lesion, and hyperplasia states. In path 1, the barrier height from the SC to the CSC state was 6.0189, and that from the CSC to cancer state was 3.5048. We can describe the carcinogenesis of SCs with a high barrier as less likely to occur, as strong regulatory and environmental conditions are required to make the SCs cancerous (Tang, 2012). With a lower barrier, differentiation of CSCs to cancer cells is an easy process, as the CSCs can generate cancer cell progeny when they divide. A CSC can be asymmetric divided into a cancer cell and another CSC (Sell, 2004). The barrier height from the SC to the normal state was 6.0509, which is comparable to the barrier from the SC to CSC state. The SC state has two choices: to become a normal differential cell or a CSC, both with certain degrees of difficulty. In adults, somatic SCs are always dormant; specific conditions are required to induce them to divide. On the other hand, reprogramming requires specific gene regulation. Therefore, the barriers for both differentiation and reprogramming are relatively high. When SCs are activated, they are asymmetric divided into SCs and normal somatic cells. It appears that in the SC state, the cell can switch to either a differentiated cell or a CSC. The paths connecting the CSC state to the SC state and the cancer state had barriers of 3.0348 and 3.5048, respectively. This illustrates that when cells stay in the CSC state, they are both very unstable owing to the low barrier height and more likely to transform to the cancer state or back to the SC state. The barrier height from the cancer to the CSC state was also high, at 9.11, which means it is difficult for cancer cells to transform back to CSCs. Experiments have revealed that only a minor proportion of cancer cells have the capacity for self-renewal and differentiation in their progeny (Liu et al., 2015). Therefore, the switching from cancer cells to CSCs is not easily realized. We can state that path 1 has the characteristics of both SCs and metastasis. Cells going through path 1 from the normal state can acquire stemness and metastasis, and eventually reach the cancer state.

In path 2, the barrier heights from the premalignant to the normal state and from the normal to the premalignant state were 3.2795 and 5.2719, respectively. The lower barrier height from the premalignant to the normal state compared with that from the normal to the premalignant state shows that it is relatively difficult for a cell in the normal state to be transformed to the premalignant state, whereas a cell in the premalignant state can relatively easily revert back to the normal state. Moreover, the barrier heights from the premalignant to the cancer state and from the cancer state to the premalignant state were 1.3395 and 7.41, respectively. This illustrates that it is much easier for a cell in the premalignant state with an intermediate level of metastasis to transform to the cancer state than the reverse from the cancer state back to the premalignant state. The barriers between the premalignant state and the normal and cancer states were lower; thus, a cell state can transform to the normal or cancer state relatively easily. The fatality of cancer is due to uncontrolled diffusion and metastasis; if cells are in the cancer state, metastasis is obvious. So, the premalignant state with an intermediate level of tumor characteristics and metastasis has vital clinical significance with respect to early diagnosis and prevention of cancer, as cells in the premalignant state can transform to cancer or revert back to a normal state easily. Therefore, path 2 has the characteristics of metastasis. Cells going through path 2 reflect the metastatic process, as the premalignant state is an intermediate state of metastasis. The importance of the premalignant state was discussed in our previous study (Yu and Wang, 2016).

In path 3, the barrier heights between the normal, lesion, and hyperplasia states were 4.0419–4.9682 and 4.7582–6.2117, that is, they were not very high. This means that it is not very difficult for cells to transform from one state to another. Experiments have shown that lesions commonly occur before hyperproliferative changes (Jeremy et al., 2003). However, the barrier heights between the hyperplasia and cancer states were 6.4117–8.11. This means that transformation from hyperplasia to cancer and the reversion from cancer to hyperplasia are both difficult, and the cancer to hyperplasia transition is unlikely to occur. That is, it is not difficult for cells to transform from one state to another before metastasis (transformation to the cancer state) occurs. If the cells have not reached metastasis, it is relatively easy for the cancer to be cured (reversion of cells to a normal state). When cells become cancerous owing to hyperplasia, this is a difficult process, but it is even more difficult to escape from the cancer state to hyperplasia as a very high barrier needs to be overcome. Therefore, path 3 reflects the process of accumulated cell damage resulting in metastasis. Cells in path 3 go through increasing pathological changes and eventually reach the cancer state.

As shown in **Figure 4**, the paths connecting the cancer state to the CSC, premalignant, and hyperplasia state had relatively high barriers of 9.11, 7.41, and 8.11, respectively. That is, the barriers of the cancer state are all very high. Thus, cells cannot easily escape the cancer state, which explains why cancer is so difficult to cure.

**Figure 5:**
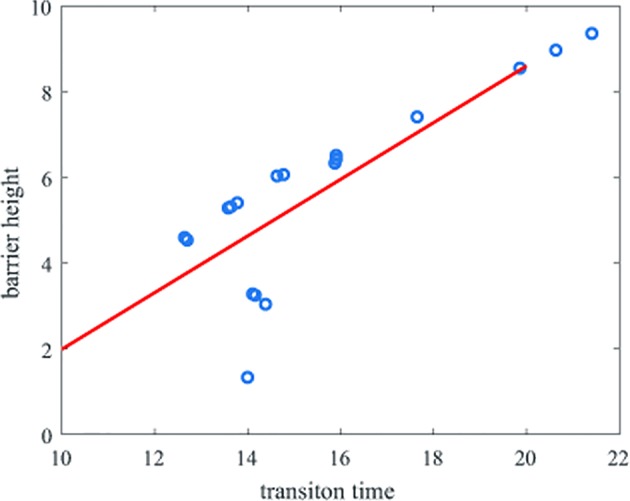
Correlation of the transition time and barrier heights. The y-axis represents the barrier height and the x-axis represents the natural logarithm of the transition time.

We also calculated the correlation of the transition time with the barrier height, obtaining a correlation coefficient of 0.80. As **Figure 5** shows, the transition time and the barrier height show almost the same trend.

We also compared the flux of the three paths (from the normal to the cancer state, and the reverse). The flux of each path indicates which path is more important in cancer formation. According to the transition time and the probability of each pathway, we could quantify the flux of each path. The transition time in our work depended on the landscape topography, which is reflected by the barrier heights of each state. The transition rate *k* of a pathway is the reciprocal of the transition time. The details and data can be seen in the **Supporting Information**. This method was used in our previous work (Wang et al., 2013). The flux of the path normal → SC → CSC → cancer was 2.2157 ∗ 10^−10^. The probability of this path was 0.0719. The flux of the path normal → premalignant → cancer was 2.6227 ∗ 10^−9^. The probability of this path was 0.8509. The flux of the path normal → lesion → hyperplasia → cancer was 2.3813 ∗ 10^−10^. The probability of this path was 0.0773. The flux and the probability of the path normal → premalignant → cancer account for the vast majority of the three. Thus, this path is dominant for the transition from the normal to the cancer state and should therefore be the focus to prevent cancer formation. We demonstrated the importance of the premalignant state for cancer prevention in our previous work (Yu and Wang, 2016).

In the same way, we could also quantify the flux from the cancer to the normal state. The flux of the path cancer → CSC → SC → normal was 2.1830 ∗ 10^−9^. The probability of this path was 0.4243. The flux of the path cancer → premalignant → normal was 9.3693 ∗ 10^−10^. The probability of this path was 0.1821. The flux of the path cancer → hyperplasia → lesion → normal was 2.0245 ∗ 10^−9^. The probability of this path was 0.3935. The flux of path 1 (cancer → CSC → SC → normal) and that of path 3 (cancer → hyperplasia → lesion → normal) were very similar, and both were higher than that of path 2 (cancer → premalignant → normal). These paths are important in the reversal of the cancer state back to the normal state. The flux and probability of path 1 were higher than those of path 3, so this path is dominant for the cancer to normal state transition. These results indicate the importance of CSCs in cancer therapy.

These three paths can be used to address a central question in cancer biology, how and which cells can be transformed to cancer, in a quantitative way. The barrier heights describe the basin depths of the landscape and help in understanding the tendency of the cells to transform from one state to another. Furthermore, the barrier heights of the cancer state are all very high, meaning that the cells in the cancer state transform less readily to others. The presence of multiple cancer formation paths explains the various mechanisms of cancer formation, which are among the reasons that cancer is difficult to prevent. The flux of the paths indicate which path is dominant in cancer formation and help to describe in a quantitative way the difficulty of curing a particular cancer.

### Finding Key Regulations by Global Sensitivity Analysis of Landscape Topography

To gain further insight into cancer formation, we explored the network to find the key regulations by global sensitivity analysis of the landscape topography. In the network, each gene and regulation contributes to the network dynamics. Variation of the regulatory strengths will influence the barrier heights between attractor basins. In this way, we could determine which regulations were more sensitive for cancer formation in the network. The results may provide a reference for drug design for cancer therapy.

**Figure 6:**
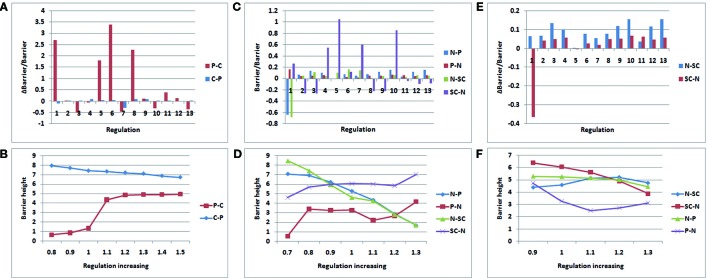
Variation rate of barrier height with regulation strength. *P* − *C* (*C* − *P*) denotes the barrier height from premalignant to cancer (cancer to premalignant) state. *N* −*P* (*P* −*N*) denotes the barrier height from normal to premalignant (premalignant to normal) state. *N* −*SC* (*SC* −*N*) denotes the barrier height from normal to SC (SC to normal) state. **(A**, **B)**:miR200┤ ZEB; **(C**, **D)**:OCT4→ OCT4; **(E**, **F)** :P53→P53.

**Figures 6A, B** display the variation of the regulation miR200˧ ZEB; regulation 1 is miR200˧ ZEB in **Figures 6A. C**, and **D** display the variation of regulation OCT4→ OCT4; regulation 1 is OCT4→ OCT4 in **Figures 6C, E**, and **F** display the variation of regulation P53→P53; regulation 1 is P53→P53 in **Figure 6E**. In **Figures 6A, C**, and **E**, the control regulations 2–13 are P53→miR200, P53→miR145, P53→MDM2, miR145˧ ZEB, miR145˧ OCT4, miR145˧ MDM2, ZEB˧ miR200, ZEB˧ miR145, ZEB→ZEB, OCT4→miR200, OCT4→miR145, and MDM2˧ P53, respectively.

As shown in **Figure 6A**, we increased the regulation strength to 1.5 times. In regulation 1 (miR200˧ ZEB), the barrier height from the premalignant state to the cancer state increased significantly, and the barrier height from the cancer state to the premalignant state decreased slightly. Although regulation 6 also changed very significantly, this was discarded as it changed in the same direction. ZEB is an EMT activator gene; when its gene expression level is high, metastasis becomes obvious. Thus, when we increased the suppression strength of ZEB, the expression level decreased, leading to weaker metastasis. In that case, it is much more difficult for the cell state to move from premalignant to cancer, and easier for it to move from cancer to premalignant, which is beneficial to cancer recovery. As shown in **Figure 6B**, we also varied the regulation strength from 0.8 to 1.5 times. When the regulation strength decreased, the barrier height from the premalignant to the cancer state also decreased, and the barrier height from the cancer to the premalignant state increased. This illustrates that the regulation is associated with the variation of the barrier height between the premalignant and cancer states. This variation of regulation miR200˧ ZEB indicates how metastasis could be controlled.

As shown in **Figure 6C**, we increased the regulation strength to 1.3 times. OCT4 is a signature gene of SCs. If the expression level of OCT4 is high, the stemness of the cell is obvious. When the regulation strength increased, the expression level of OCT4 increased, the barrier height from the normal to the SC state decreased, and the barrier height from the SC to the normal state increased significantly. This means that a cell in the normal state could more easily move to the SC state, but it was more difficult for cells in the SC state to move to the normal state. At the same time, the barrier height from the normal to the premalignant state decreased, and that from the premalignant to the normal state increased. This indicates that a cell in the normal state is more likely to become cancerous, and a cell in the premalignant state is less likely to move back. These results are consistent with those of experiments involving iPS. Many studies have shown that the cellular reprogramming of iPS often leads to cells with cancerous characteristics, which eventually reach the cancer state (Kondo and Raff., 2000; Pavlova and Thompson, 2016). As shown in **Figure 6D**, when the regulation strength decreased to 0.7 times, the barrier height from the normal to the premalignant state increased, and that from the premalignant to the normal state decreased. This indicates that the cells in the normal state are more stable, and those in the premalignant state are more likely to transform to the normal state. The barrier height from the normal to the SC state increased, and that from the SC to the normal state decreased. Thus, cells in the normal state are less likely to switch to the SC state, and those in the SC state are more apt to transform to the normal state. The regulation OCT4→OCT4 reflects the connection between SCs and metastasis. This may guide the search for cancer treatments involving SCs.

As shown in **Figure 6E**, we also increased the regulation strength to 1.3 times. The variation of regulation 1 (P53→P53) increased the barrier height from the normal to the SC state, and decreased that from the SC to the normal state. This indicates that when the expression level of P53 increases, the cells in the normal state are less likely to transform to the SC state, whereas the cells in the SC state were more likely to transform to the normal state. Experiments have shown that P53 is a major driving force for the differentiation of embryonic SCs (ESCs). Spontaneous differentiation of hESCs reduced significantly when P53 expression decreased (Qin et al., 2007). P53 also can provide an effective barrier for the generation of stemness cells from terminally differentiated cells (Solozobova and Blattner, 2011). The variation of regulation P53→P53 illustrates the importance of P53 not only for cancer but also for SC processes.

P53 is a tumor suppressor, as shown in **Figure 6F**. When the regulation strength decreased to 0.9 times and P53 abundance was reduced, the barrier height from the normal to the premalignant state barely changed, but the barrier height from the premalignant to the normal state increased significantly. In this situation, the cells in the premalignant state are less likely to move back to the normal state. When the regulation strength was increased to 1.1 times, the barrier height from the normal to the premalignant state showed no significant change, whereas that from the premalignant to the normal state was reduced. That means the cells in the premalignant state would more easily transition to the normal state. When the regulation strength was increased to 1.2 and 1.3 times, the barrier height between the normal and premalignant states varied only slightly. When the concentration of P53 reaches a very high level, its tumor suppressor characteristics become less obvious and other characteristics are present, such as inducing apoptosis (Haupt et al., 2003).

**Figure 7:**
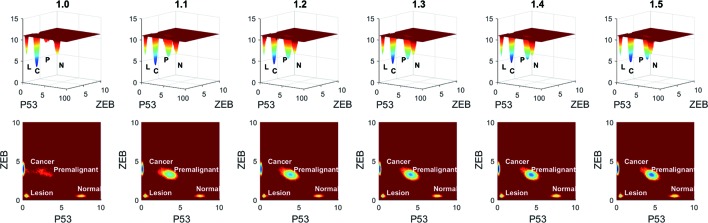
Variation of barrier height when the regulation strength changes from 1.0 to 1.5 times. *L* represents the lesion state, *P* represents the premalignant state, *C* represents the cancer state, and *N* represents the normal state.

To see the variation more clearly, we depicted the landscape topography of miR200┤ ZEB from regulation strength 1.0 to 1.5 times. As shown in **Figure 7**, the depth of the basin of the premalignant state increased significantly when the regulation strength increased, and the depth of the basin of the cancer state decreased.

## Conclusions

Cancer is a complex and fatal disease. Its features of metastasis, drug resistance, and recurrence, which are related to CSCs, cause cancer to be a major health threat. Recent studies have shown that EMT has a vital role in inducing the early stage of metastasis, as well as being a way for non-SCs to transform into SCs. In this study, we developed a dynamic model which includes specific genes and microRNAs for CSC, EMT, and cancer, with the aim of uncovering the connections among cancer, metastasis, and differentiation and development in CSC, EMT, and cancer. We quantified the underlying landscape to explore differentiation and development and metastasis, thereby elucidating the origin of cancer. The kinetic paths and barrier heights between each state were quantified. The barrier heights determine the stability of the state and relate to the switching frequency of the cells from one state to another. Multiple cancer formation pathways were observed. The flux of each path (from normal to cancer, and the reverse) was calculated using the statistics of the path transitions. This was used to determine which path is more important in cancer formation and treatment, and could also help to quantify the degree of difficulty of curing a particular cancer. Furthermore, we used global sensitivity analysis to find key regulations which are vital for cancer formation. Three regulations, miR200┤ ZEB, OCT4→ OCT4, and P53→P53 were more sensitive than other regulations. These regulations may provide a reference for the treatment of cancer. This work studied the functional dynamics and physical mechanisms of differentiation and development in cancer and metastasis in a quantitative way, and may serve as a guide for clinical therapy of cancer.

## Data Availability Statement

Publicly available datasets were analyzed in this study. This data can be found in TCGA-LIHC and TCGA-COAD projects.

## Ethics Statement

The authors declare that the study doesn't include animal and human experiments that violate ethics.

## Author Contributions

Article writing by CY and JW. Model constructed by CC, CY, QL, and JW. Data analysis by CY, QL, and JW. Analyzing tools provided by JW.

## Funding

CY and QL were supported by NSFC grant no. 21721003, MOST China grant no. 2016YFA0203200. JW thanks NSF-PHY-76066 and NSF-CHE1808474 for supports.

## Conflict of Interest

The authors declare that the research was conducted in the absence of any commercial or financial relationships that could be construed as a potential conflict of interest.
