# Supplementary material for: Quantification of the Underlying Mechanisms and Relationships Among Cancer, Metastasis, and Differentiation and Development
Source: Front Genet. 2020 Mar 2;10:1388. doi: 10.3389/fgene.2019.01388 (PMC7061528; doi:10.3389/fgene.2019.01388)
Supplement: Supplementary file 1 [file DataSheet_1.docx]

**Supporting information of**

**Quantification of the underlying mechanisms and relationship among**

**cancer, metastasis and differentiation/development**

**Chong Yu,^1;2^Qiong Liu,^1^Cong Chen,^3^and Jin Wang^3∗^**

**^1^State Key Laboratory of Electroanalytical Chemistry**

**Changchun Institute of Applied Chemistry, Chinese Academy of Sciences**

**Changchun, Jilin 130022, China**

**^2^University of Science and Technology of China**

**^3^Department of Chemistry and of Physics and Astronomy**

**State University of New York at Stony Brook**

**Stony Brook, NY 11794-3400, USA**

**^∗^Corresponding Authors:** [**jin.wang.1@stonybrook.edu**](mailto:jin.wang.1@stonybrook.edu)

## Table S1：Literature search results, *a* represents activation, *r* represents repression.

| *TP53* | *MDM2* | *a* | *[*[*1*](#_ENREF_1)*]* |
| --- | --- | --- | --- |
| *TP53* | *TP53* | *a* | *[*[*2*](#_ENREF_2)*]* |
| *TP53* | *miR-200* | *a* | *[*[*3*](#_ENREF_3)*]* |
| *MDM2* | *TP53* | *r* | *[*[*4*](#_ENREF_4)*]* |
| *OCT4* | *OCT4* | *a* | *[*[*5*](#_ENREF_5)*]* |
| *OCT4* | *miR-200* | *a* | *[*[*6*](#_ENREF_6)*]* |
| *OCT4* | *miR-145* | *r* | *[*[*7*](#_ENREF_7)*]* |
| *miR-145* | *OCT4* | *r* | *[*[*8*](#_ENREF_8)*]* |
| *miR-145* | *MDM2* | *r* | *[*[*9*](#_ENREF_9)*]* |
| *miR-145* | *ZEB* | *r* | *[*[*10*](#_ENREF_10)*]* |
| *ZEB* | *ZEB* | *a* | *[*[*11*](#_ENREF_11)*,* [*12*](#_ENREF_12)*]* |
| *ZEB* | *miR-200* | *r* | *[*[*13*](#_ENREF_13)*]* |
| *ZEB* | *miR-145* | *r* | *[*[*12*](#_ENREF_12)*]* |
| *miR-200* | *ZEB* | *r* | *[*[*13*](#_ENREF_13)*]* |

In the table, *a* represents the activation regulation, and *r* represents the repression regulation.

## Table S2:Gene functions

| **Gene** | **Function** |
| --- | --- |
| TP53 | Tumor suppressor gene[[14](#_ENREF_14)] |
| MDM2 | Oncogene, tumor suppressor gene[[15](#_ENREF_15)] |
| ZEB | ZEB1 and ZEB2 are crucial regulators of EMT during embryonic development and cancer.[[16](#_ENREF_16)] |
| OCT4 | maintaining the pluripotency and self-renewal of stem cells[[17](#_ENREF_17)] |
| mir-200 | Up-regulated mir-200 and down-regulated mir-145 can increase self-renewal and migration.[[18](#_ENREF_18)] |
| mir-145 |  |

## Quantification of potential landscape and determination of barrier heights between basins

The 3D histogram of P53, ZEB and OCT4 expression can be constructed using time traces obtained by the simulation of Gillespie Algorithm.And it was also the probability landscape *p* with respect to P53, ZEB and OCT4 expression. The potential landscape U is closely related to the probability landscape by the relationship U = -ln(*p*).

From the quantified potential landscape, we identified seven basins of attractions (the location of the basin is defined as the location of the potential landscape) and the barrier heights between these basins. Here, the barrier heights(for example, from basin A to basin B) are defined as the difference between the potential of basin A and the saddle point value between the two basins. There are many paths from basin A to basin B on the potential landscape. The maximum potential value is the barrier of the corresponding path. The most possible path is correspond to the minimum value of the barrierof all the possible paths, that is the saddle point value of the landscape from basin A to basin B on the potential landscape. Thesaddle point values are found by Genetic Algorithm.The fitness function of Genetic Algorithm is the maximum value of each possible path.The initial path is a random path between the selected starting and ending point. Due to the discontinuous of the point between basins, we get the continuouspotential by linear interpolation. By modifying the parameters according to the Genetic Algorithm, we can get the optimal saddle point value of each transition. The specific process if as follows:

1. Find the center of mass of the basin A and basin B. Take the center of mass position as the starting and ending point of the trial path.
2. The random path generation. Start at the starting point and find a path from basin A to basin B by self-avoiding walk. Find 20 initial paths using the self-avoiding walk.
3. Calculate the fitness value of all the initial path. The fitness value is the maximum value of each possible path. If the path pass through any state other than basin A and basin B, then the fitness value will be multiplied by a large penalty value (for example 100)
4. Generate new path by crossing the two random initial paths.
5. Generate new path by mutation the initial path.
6. Calculate all the fitness value of these new paths and initial paths. Normalize all fitness values as probabilities, and then randomly select 20 paths as the initial paths.
7. Go to step 3 until reach the number of generations. Give the optimal saddle point value of each transition. The saddle point value is the barrier height potential.
8. The barrier height is the difference between the barrier height potential obtained in step (7) and the potential of each state.
9. **Calculation of Residence time and transition number**

From the quantified potential landscape, we identified seven basins of attractions and their location (the location of the basin is defined as the location of the potential landscape). The trajectory of P53, ZEB and OCT4 expression can be obtained by Gillespie Algorithm simulation. The state sequence of the trajectory can be also distinguished by the location of the potential landscape. The residence time is the average transition time from one state to another state. The transition number is the total transition frequency for all simulation trajectories. The probability transition matrix is the normalized transition number for each row. The specific process if as follows:

1. Calculate the state at each point in time along the trajectory.
2. Select two states to calculate, the starting state and the ending state.
3. Go through the entire trajectory; find all the transitions from the starting state to the ending state. The number of transitions found above is the transition number for the starting state and the ending state.
4. For all the transitions found above, count all the duration time of the starting state. The average value of all the duration time is the residence time for the starting state and the ending state.
5. Go to step 2 for another pair of states, until all the state pairs are done.
6. **Calculation of the pathway flux**

The pathway flux is proportional to the state probability in the pathway and the transition rates. The transition probabilities are correlated to the transition rates. Because there are multiple kinetic paths to transition between two states, the transition rate is the apparent value of these paths. To identify transition rates between every two states, we used the master equation approach. The master equation can be written as

$\frac{d}{dt}\left(\begin{matrix} P_{1} \\ P_{2} \\ P_{3} \\ \begin{matrix} P_{4} \\ P_{5} \\ P_{6} \\ P_{7} \end{matrix} \end{matrix}\right)=\left[\begin{matrix} k_{11} & k_{12} & k_{13} & k_{14} & k_{15} & k_{16} & k_{17} \\ k_{21} & k_{22} & k_{23} & k_{24} & k_{25} & k_{26} & k_{27} \\ k_{31} & k_{32} & k_{33} & k_{34} & k_{35} & k_{36} & k_{37} \\ k_{41} & k_{42} & k_{43} & k_{44} & k_{45} & k_{46} & k_{47} \\ k_{51} & k_{52} & k_{53} & k_{54} & k_{55} & k_{56} & k_{57} \\ k_{61} & k_{62} & k_{63} & k_{64} & k_{65} & k_{66} & k_{67} \\ k_{71} & k_{72} & k_{73} & k_{74} & k_{75} & k_{76} & k_{77} \end{matrix}\right]\left(\begin{matrix} P_{1} \\ P_{2} \\ P_{3} \\ \begin{matrix} P_{4} \\ P_{5} \\ P_{6} \\ P_{7} \end{matrix} \end{matrix}\right)$(1)

Where P_i_, i=1,2,3,4,5,6,7 are the probabilities of states determined by the depth of the basin, respectively. k_ij_,(i, j=1,2,3,4,5,6,7) is the transition rate from P_j_ to P_i_. Due to the probability conservation, k_11_=-(k_21_+k_31_+k_41_+k_51_+k_61_+k_71_). Similar results can be obtained for the other states:

k_22_=-(k_12_+k_32_+k_42_+k_52_+k_62_+k_72_)

k_33_=-(k_13_+k_23_+k_43_+k_53_+k_63_+k_73_)

k_44_=-(k_14_+k_24_+k_34_+k_54_+k_64_+k_74_)

k_55_=-(k_15_+k_25_+k_35_+k_45_+k_65_+k_75_)

k_66_=-(k_16_+k_26_+k_36_+k_46_+k_56_+k_76_)

k_77_=-(k_17_+k_27_+k_37_+k_47_+k_57_+k_67_)

Then, we can substitute these above formula into the master equation, and get the result of minimum parameters.

Once we know the values of the rate matrix of the master equation and the initial probabilities of each state, the transition probability can be directly quantified. Our goal is to deduce the transfer rate in the master equation from the known transition probability matrix. This process is implemented as follows. Through the numerical solution of the master equation, we can get the probability of unit time, this probability corresponds to an element of the transition probability matrix, for example, when P_1_=1, and the others are all zero. The calculation of the seven probabilities are the transition probabilities from P_1_ to P_j_ (j=1,2,3,4,5,6,7), respectively. Therefore, for given set of the rate parameters, the corresponding transition probability matrix can be obtained by using different initial values (one of state probability is 1, and the others are all zero). In this way, we can establish the connections between the rate matrix in master equation and the transition probability matrix obtained by Gillespie simulation. Using Genetic Algorithm, we can find the optimal parameters of the rate matrix, and we use these rate values to calculate the pathway flux.

1. **Gillespie simulation**

We use Gillespie Algorithm to obtain the stochastic distribution time of protein binding/unbinding. In a bio-chemical system, reactions will happen anytime. So the variations of the system states must be a complex process of dynamics. There are M reactions {R_1_,R_2_,...,R_M_} of the protein regulations. Each reaction R_μ_ corresponds to a propensity function a_μ_. a_μ_(x)dt is defined as: to a given X(t)=x, the probability of R_μ_ has reacted once within time [t,t+dt). In general, it is hard to describe the propensity function a_μ_ accurately. We can obtain an approximate description: $a_{\mu}\left( x \right)=c_{\mu}\prod_{k=1}^{N} C_{x_{k}}^{m_{\mu_{k}}}$. $c_{\mu}\prod_{k=1}^{N} C_{x_{k}}^{m_{\mu_{k}}}$ is the probability of the molecular S_k_ will participate in the reaction R_μ_. The value of x_k_ represents total number of S_k_, and m_k_ represents the number of reactants which participate in the reaction. c_μ_ is the constant of chemical reaction, which can obtain from experiments. Due to intrinsic fluctuations and variations of the protein molecule numbers in the cells, we explore the stochastic dynamics time of each protein binding/unbinding steps and change the protein molecule numbers correspondingly as $c_{\mu}\prod_{k=1}^{N} C_{x_{k}}^{m_{\mu_{k}}}$.

The probability density function of the system can be described as: $P\left( \tau,\mu;x \right)=P_{0}(\tau,x)\bullet a_{\mu}(x)d\tau$.$P_{0}(\tau,x)$ represents the probability of a specified system will not react within a time interval (t,t+τ) . $a_{\mu}(x)d\tau$represents the probability of reaction R_μ_ reacted within a time interval (t+τ,t+τ+ dτ).

The main steps of the algorithm are as follows:

1. Initialization: $X\left( 0 \right)=x_{0}$, and set the initial time t=0.
2. Calculate the reaction rate $a_{\mu}$=$a_{\mu}\left( x \right)(\mu=1,\ldots,M)$， and set $a_{0}$=$\sum_{\mu=1}^{M} a_{\mu}$.$a_{\mu}$is the chemical reaction rate of each reaction $R_{\mu}$.
3. Generate random pair (τ,μ), according to the probability density function

P(τ,μ)=$\left\{ \begin{aligned} a_{\mu}e^{-a_{0}{(x)}^{\tau}}, if 0\leq\tau\leq\infty and \mu=1,\ldots,M \\ 0,others \end{aligned} \right.$

1. Set t=t+τ,update the molecular numbers, according to the reaction$R_{\mu}$,$X_{i}\to X_{i}+v_{\mu i.}$
2. Return to step 2.

The simulation results will be further validated by the experimental data. If the results are consistent with the experimental data, the model will be successfully constructed. Otherwise, it shows that the model is still deviated from the actual situation and will continue to adjust the model.

1. **The regulation of the three group genes**

*1.Cancer group: the character ‘a’ represents activation, ‘r’ represents repression.*

| P53 | 'MDM2' | a | [[1](#_ENREF_1)] |
| --- | --- | --- | --- |
|  | 'BRCA1' | r | [[19](#_ENREF_19)] |
|  | 'BRCA2' | r | [[20](#_ENREF_20)] |
|  | 'ERBB2' |  | P53-EGFR-REBB2 |
|  | 'ATR' | a | P53->BRCA1->ATR |
|  | 'E2F1' | r | [[21](#_ENREF_21)] |
|  | 'CDK2' | r | P53-\|P21->CDK2 |
|  | 'CHEK1' | r | [[22](#_ENREF_22)] |
|  | 'CHEK2' | r | [[23](#_ENREF_23)] |
|  | 'ATM' | a | [[24](#_ENREF_24)] |
|  | 'AKT1' | r | [[25](#_ENREF_25)] |
|  | 'PTEN' | a | [[26](#_ENREF_26)] |
|  | 'EGFR' | a | [[27](#_ENREF_27)] |
|  | 'NFKB1' | r | [[28](#_ENREF_28)] |
|  | 'NFKB2' | r | [[28](#_ENREF_28)] |
|  | 'BAX' | r | [[29](#_ENREF_29)] |
|  | 'BAD' | a | [[30](#_ENREF_30)] |
|  | 'CDK1' | a | [[31](#_ENREF_31)] |

*2. Stemcell group*

| OCT4 | 'NANOG' | a | [[32](#_ENREF_32)] |
| --- | --- | --- | --- |
|  | 'SOX2' | a | [[33](#_ENREF_33)] |
|  | 'GATA6' | r | [[34](#_ENREF_34)] |
|  | 'KLF4' | a | [[33](#_ENREF_33)] |
|  | 'GDF3' | a | OCT4-TGFβ-GDF3 |
|  | 'ZIC3' |  | [[35](#_ENREF_35)] |
|  | 'PBX1' | a | OCT4- TGFβ-PBX1 |
|  | 'TDGF1' | a | [[36](#_ENREF_36)] |
|  | 'ZFP42' |  | [[37](#_ENREF_37)] |
|  | 'FOXD3' | a | [[38](#_ENREF_38)] |

*3. EMT group*

| ZEB | 'HIF1A' | a |  |
| --- | --- | --- | --- |
|  | 'MIR200A' | r | [[39](#_ENREF_39)] |
|  | 'MIR200B' | r | [[40](#_ENREF_40)] |
|  | 'MIR200C' | r | [[39](#_ENREF_39)] |
|  | 'MIR141' | r | [[40](#_ENREF_40)] |
|  | 'MIR429' |  | ZEB- MIR200A - MIR429 |
|  | 'MIR34C' | r | ZEB- MIR141 – MIR34C |
|  | 'MIR34B' | r | ZEB- MIR141 – MIR34B |
|  | 'BACH1' | a | ZEB- HIF1A - BACH1 |
|  | 'LIN28' |  | ZEB- MIR145 – LIN28 |
|  | 'HGF' |  | ZEB- HIF1A - HGF |

**References**

1. Freedman, D.A., L. Wu, and A.J. Levine, *Functions of the MDM2 oncoprotein.* Cellular and Molecular Life Sciences, 1999. **55**(1): p. 96-107.

2. Sanduja, S., V. Kaza, and D.A. Dixon, *The mRNA decay factor tristetraprolin (TTP) induces senescence in human papillomavirus-transformed cervical cancer cells by targeting E6-AP ubiquitin ligase.* Aging, 2009. **1**(9): p. 803-817.

3. Chang, C.J., et al., *p53 regulates epithelial-mesenchymal transition and stem cell properties through modulating miRNAs (vol 13, pg 317, 2011).* Nature Cell Biology. **13**(12): p. 1466-1466.

4. Shinozaki, T., et al., *Functional role of Mdm2 phosphorylation by ATR in attenuation of p53 nuclear export.* Oncogene, 2003. **22**(55): p. 8870-8880.

5. Kim, J.H., et al., *Regulation of adipose tissue stromal cells behaviors by endogenic Oct4 expression control.* Plos One, 2009. **4**(9): p. e7166.

6. Pandey, A., et al., *Critical role of the miR-200 family in regulating differentiation and proliferation of neurons.* Journal of Neurochemistry. **133**(5): p. 640-652.

7. Liu, T., et al., *Curcumin suppresses proliferation and in vitro invasion of human prostate cancer stem cells by ceRNA effect of miR-145 and lncRNA-ROR.* Gene. **631**: p. 29-38.

8. Xu, N., et al., *MicroRNA-145 Regulates OCT4, SOX2, and KLF4 and Represses Pluripotency in Human Embryonic Stem Cells.* Cell, 2009. **137**(4): p. 647-658.

9. Zhang, J., et al., *Loss of microRNA-143/145 disturbs cellular growth and apoptosis of human epithelial cancers by impairing the MDM2-p53 feedback loop.* Oncogene. **32**(1): p. 61-69.

10. Polytarchou, C., D. Iliopoulos, and K. Struhl, *An integrated transcriptional regulatory circuit that reinforces the breast cancer stem cell state.* Proceedings of the National Academy of Sciences of the United States of America. **109**(36): p. 14470-14475.

11. Orit, L., et al., *TMPRSS2/ERG promotes epithelial to mesenchymal transition through the ZEB1/ZEB2 axis in a prostate cancer model.* Plos One. **6**(7): p. e21650.

12. Kolesnikoff, N., et al., *Specificity Protein 1 ( Sp1) Maintains Basal Epithelial Expression of the miR-200 Family.* Journal of Biological Chemistry. **289**(16): p. 11194-11205.

13. Lu, M., et al., *MicroRNA-based regulation of epithelial-hybridmesenchymal fate determination.* Proceedings of the National Academy of Sciences of the United States of America. **110**(45): p. 18144-18149.

14. Maruei-Milan, R., Z. Heidari, and S. Salimi, *Role of MDM2 309T>G (rs2279744) and I/D (rs3730485) polymorphisms and haplotypes in risk of papillary thyroid carcinoma, tumor stage, tumor size, and early onset of tumor: A case control study.* Journal of cellular physiology. **234**(8): p. 12934-12940.

15. Elkholi, R., et al., *MDM2 Integrates Cellular Respiration and Apoptotic Signaling through NDUFS1 and the Mitochondrial Network.* Molecular cell. **74**(3): p. 452-465.e7.

16. Das, S., et al., *Complete reversal of epithelial to mesenchymal transition requires inhibition of both ZEB expression and the Rho pathway.* Bmc Cell Biology, 2009. **10**.

17. Chen, Y., et al., *OCT4B-190 protects against ischemic stroke by modulating GSK-3beta/HDAC6.* Experimental neurology. **316**: p. 52-62.

18. Mashayekhi, P., et al., *Endometriotic Mesenchymal Stem Cells Epigenetic Pathogenesis: Deregulation of miR-200b, miR-145, and let7b in A Functional Imbalanced Epigenetic Disease.* Cell Journal. **21**(2): p. 179-185.

19. Kranz, D., C. Dohmesen, and M. Dobbelstein, *BRCA1 and Tip60 determine the cellular response to ultraviolet irradiation through distinct pathways.* Journal of Cell Biology, 2008. **182**(1): p. 197-213.

20. Gochhait, S., et al., *Implication of BRCA2-26G > A 5 ' untranslated region polymorphism in susceptibility to sporadic breast cancer and its modulation by p53 codon 72 Arg > Pro polymorphism.* Breast Cancer Research, 2007. **9**(5).

21. Taura, M., et al., *MEF/ELF4 transactivation by E2F1 is inhibited by p53.* Nucleic Acids Research, 2011. **39**(1): p. 76-88.

22. Cummings, M., et al., *p53-mediated downregulation of Chk1 abrogates the DNA damage-induced G2M checkpoint in K562 cells, resulting in increased apoptosis.* British Journal of Haematology, 2002. **116**(2): p. 421-428.

23. Matsui, T., et al., *Negative regulation of Chk2 expression by p53 is dependent on the CCAAT-binding transcription factor NF-Y.* Journal of Biological Chemistry, 2004. **279**(24): p. 25093-25100.

24. Fujita, M., *Cdt1 revisited: complex and tight regulation during the cell cycle and consequences of deregulation in mammalian cells.* Cell Division, 2006. **1**.

25. Bachelder, R.E., et al., *p53 inhibits alpha 6 beta 4 integrin survival signaling by promoting the caspase 3-dependent cleavage of AKT/PKB.* Journal of Cell Biology, 1999. **147**(5): p. 1063-1072.

26. Steelman, L.S., et al., *Roles of the Raf/MEK/ERK and PI3K/PTEN/Akt/mTOR pathways in controlling growth and sensitivity to therapy-implications for cancer and aging.* Aging-Us, 2011. **3**(3): p. 192-222.

27. Klanrit, P., et al., *PML involvement in the p73-mediated E1A-induced suppression of EGFR and induction of apoptosis in head and neck cancers.* Oncogene, 2009. **28**(39): p. 3499-3512.

28. Ghose, J., et al., *Regulation of miR-146a by RelA/NFkB and p53 in STHdh(Q111)/Hdh(Q111) Cells, a Cell Model of Huntington's Disease.* Plos One, 2011. **6**(8).

29. Verma, S., M.M. Tabb, and B. Blumberg, *Activation of the steroid and xenobiotic receptor, SXR, induces apoptosis in breast cancer cells.* Bmc Cancer, 2009. **9**.

30. Wee, K.B., U. Surana, and B.D. Aguda, *Oscillations of the p53-Akt Network: Implications on Cell Survival and Death.* Plos One, 2009. **4**(2).

31. Gil-Gomez, G., A. Berns, and H.J.M. Brady, *A link between cell cycle and cell death: Bax and Bcl-2 modulate Cdk2 activation during thymocyte apoptosis.* Embo Journal, 1998. **17**(24): p. 7209-7218.

32. Noisa, P. and R. Parnpai, *Technical Challenges in the Derivation of Human Pluripotent Cells.* Stem Cells International, 2011.

33. Kim, J.H., et al., *Regulation of Adipose Tissue Stromal Cells Behaviors by Endogenic Oct4 Expression Control.* Plos One, 2009. **4**(9).

34. Yeap, L.-S., K. Hayashi, and M.A. Surani, *ERG-associated protein with SET domain (ESET)-Oct4 interaction regulates pluripotency and represses the trophectoderm lineage.* Epigenetics & Chromatin, 2009. **2**.

35. Lim, L.S., et al., *Zic3 is required for maintenance of pluripotency in embryonic stem cells.* Molecular Biology of the Cell, 2007. **18**(4): p. 1348-1358.

36. Marikawa, Y., et al., *Dual Roles of Oct4 in the Maintenance of Mouse P19 Embryonal Carcinoma Cells: As Negative Regulator of Wnt/beta-Catenin Signaling and Competence Provider for Brachyury Induction.* Stem Cells and Development, 2011. **20**(4): p. 621-633.

37. Matoba, R., et al., *Dissecting Oct3/4-Regulated Gene Networks in Embryonic Stem Cells by Expression Profiling.* Plos One, 2006. **1**(1).

38. Chavez, L., et al., *In silico identification of a core regulatory network of OCT4 in human embryonic stem cells using an integrated approach.* Bmc Genomics, 2009. **10**.

39. Cochrane, D.R., et al., *Loss of miR-200c: A Marker of Aggressiveness and Chemoresistance in Female Reproductive Cancers.* Journal of oncology, 2010. **2010**: p. 821717-821717.

40. Cho, J.-H., et al., *Systems biology of interstitial lung diseases: integration of mRNA and microRNA expression changes.* Bmc Medical Genomics, 2011. **4**.
